# Identification and characterization of microRNAs involved in growth of blunt snout bream (*Megalobrama amblycephala*) by Solexa sequencing

**DOI:** 10.1186/1471-2164-14-754

**Published:** 2013-11-05

**Authors:** Shaokui Yi, Ze-Xia Gao, Honghao Zhao, Cong Zeng, Wei Luo, Boxiang Chen, Wei-Min Wang

**Affiliations:** 1 College of Fisheries, Key Lab of Agricultural Animal Genetics, Breeding and Reproduction of Ministry of Education/Key Lab of Freshwater Animal Breeding, Ministry of Agriculture, Huazhong Agricultural University, Wuhan, Hubei, 430070, China; 2 Freshwater Aquaculture Collaborative Innovation Center of Hubei Province, Wuhan, 430070, China; 3 Hu Bei Bai Rong Improved Aquatic Seed CO., LTD, Huanggang, Hubei, 438800, China; 4 School of Biological Science, Victoria University of Wellington, Wellington, 6014, New Zealand

## Abstract

**Background:**

Blunt snout bream (*Megalobrama amblycephala*) is an economically important fish species in the Chinese freshwater polyculture system for its delicacy and high economic value. MicroRNAs (miRNAs) play important roles in regulation of almost all biological processes in eukaryotes. Although previous studies have identified thousands of miRNAs from many species, little information is known for miRNAs of *M. amblycephala*. To investigate functions of miRNAs associated with growth of *M. amblycephala*, we adopted the Solexa sequencing technology to sequence two small RNA libraries prepared from four growth related tissues (brain, pituitary, liver and muscle) of *M. amblycephala* using individuals with relatively high and low growth rates.

**Results:**

In this study, we have identified 347 conserved miRNAs (belonging to 123 families) and 22 novel miRNAs in *M. amblycephala*. Moreover, we observed sequence variants and seed edits of the miRNAs. Of the 5,166 single nucleotide substitutions observed in two libraries, the most abundant were G-to-U (15.9%), followed by U-to-C (12.1%), G-to-A (11.2%), and A to G (11.2%). Subsequently, we compared the expression patterns of miRNAs in the two libraries (big-size group with high growth rate versus small-size group with low growth rate). Results indicated that 27 miRNAs displayed significant differential expressions between the two libraries (*p* < 0.05). Of these, 16 were significantly up-regulated and 11 were significantly down-regulated in the big-size group compared to the small-size group. Furthermore, stem-loop RT-PCR was applied to validate and profile the expression of the differentially expressed miRNAs in ten tissues, and the result revealed that the conserved miRNAs expressed at higher levels than the novel miRNAs, especially in brain, liver and muscle. Also, targets prediction of differentially expressed miRNAs and KEGG pathway analysis suggested that differentially expressed miRNAs are involved in growth and metabolism, signal transduction, cell cycle, neural development and functions.

**Conclusions:**

The present study provides the first large-scale characterization of miRNAs in *M. amblycephala* and miRNA profile related to different growth performances. The discovery of miRNA resource from this study is expected to contribute to a better understanding of the miRNAs roles playing in regulating the growth biological processes and the study of miRNA function and phenotype-associated miRNA identification in fish.

## Background

Blunt snout bream (*Megalobrama amblycephala* Yih, 1955), which natural distribution is limited in the middle and lower reaches of the Yangtze River in China [1], has been widely favored for its delicacy and recognized as a main aquaculture species in the polyculture system of Chinese freshwater fish since 1960s [2]. Due to its high economic value, the total production of *M. amblycephala* is rapidly growing [3]. However, owing to overfishing of wild resources, artificial breeding and fast domestication, the germplasm resources of *M. amblycephala* are under threat of recession and admixture [4]. Unfortunately, the cultured population of *M. amblycephala* gradually exhibits growth depression, early sexual maturity, and disease susceptibility. At present, molecular techniques are still not widely used in the breeding of *M. amblycephala* due to a lack of genetic and genomic information. In recent years, several traits of *M. amblycephala*, including body shape, hypoxia resistance and disease resistance, have been studied through development of molecular markers [4-6]. Although it is an important economic trait, the growth rate of *M. amblycephala* has not been well defined by molecular markers or control mechanisms.

MicroRNAs (miRNAs) are a class of small (approximately 22 nucleotides; nt) endogenous noncoding RNAs in length [7], which are embedded within the stem regions of hairpin transcripts that exist in a wide range of invertebrates and vertebrates. miRNAs play a pivotal role in the regulation of gene expression at the post-transcriptional level, especially for signaling pathways involved in development, cellular differentiation, proliferation, apoptosis, and oncogenesis [8]. They negatively regulate gene expression through sequence-specific interactions with the 3′ untranslated regions (UTRs) of target mRNAs and thereby cause translational repression or mRNA destabilization [8,9]. Since the discovery of the founding members of the miRNA family, lin-4 and let-7 in *Caenorhabditis elegans* in 1993 [10-12], many endogenously encoded miRNAs have been identified in mammals, plants, insects, worms, and viruses through plasmid vector cloning, northern blotting, microarray assay and sequencing technology in recent years [13-15]. Currently, 21264 mature miRNAs from 193 species have been discovered and deposited in the public available miRNA database miRBase (Release 19.0, June 2013) [16].

Recently, next-generation sequencing technology has a substantial impact on a broad range of biological applications. Sequencing technology made it possible to precisely identify non-conserved or weakly expressed miRNAs, and been widely used to facilitate the identification and detection of miRNAs in multiple species, such as fish, chicken and silkworm [14,17,18]. Previous studies have identified many miRNAs from model fish species [19]; nonetheless, little attention has been given to the miRNAs’ roles in non-model species. The identification of miRNAs in aquaculture fish species began with the study of cloning and characterization of miRNAs from the rainbow trout (*Oncorhynchus mykiss*) in 2008 [20]. Subsequently, a large number of miRNAs has been identified in other aquaculture species, including the Japanese flounder (*Paralichthys olivaceus*) [21], bighead carp (*Hypophthalmichthys nobilis*), silver carp (*H. molitrix*) [22], common carp (*Cyprinus carpio*) [23] and channel catfish (*Ictalurus punctatus*) [24]. Unfortunately, little information is available in *M. amblycephala*. In this study, we constructed two small-RNA cDNA libraries from the growth related tissues (brain, pituitary, liver and muscle) of *M. amblycephala* using individuals with relatively high and low growth rates. Through high throughput sequencing of the small RNA library and subsequent bioinformatic analysis, miRNAs in two libraries of *M. amblycephala* were identified and the differentially expressed miRNAs were analyzed. The discovery of miRNA resource from this study will contribute to a better understanding of the miRNAs roles playing in regulating the growth biological processes in fish and the study of miRNA function and phenotype-associated miRNA identification in *M. amblycephala*.

## Result and discussion

### Solexa sequencing of small RNAs

**Figure 1:**
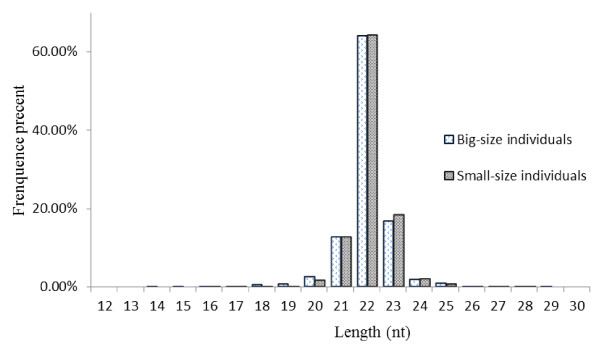
**Length distribution of small RNAs in *M. amblycephala*.**

**Figure 2:**
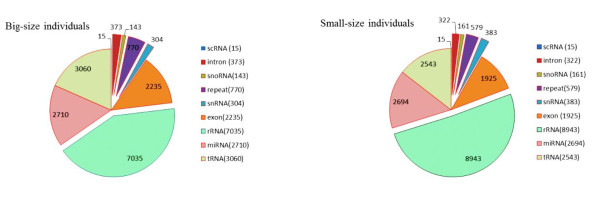
**Annotation of small RNAs derived from Solexa sequencing of *M. amblycephala *** **small RNAs libraries.**

In order to identify the miRNAs involved in the growth rate of *M. amblycephala*, two small RNA libraries were constructed, with the mixed pools of tested tissues (brain, pituitary, liver and muscle) from the big-size group and small-size group with relatively high and low growth rates at 3, 6, 12 and 18-month-old stages, respectively. Through high throughput Solexa sequencing, a total of 19,144,188 high quality reads were obtained. Very little difference was found in the length distribution of the sequences from the two libraries, most of the sequences (94.28%) being between 21–23 nucleotides (Figure 1). After removal of the adapters, reads with polyA and reads smaller than 18 nucleotides, 19,069,714 clean reads were extracted. The total number of unique clean reads from big-size and small-size groups sRNA libraries was 117,169 and 99,070, respectively (Additional file 1: Table S1-1). These unique sequences contained 26,784 common sequences between the big-size and small-size libraries (Additional file 1: Table S1-2). After comparing the small RNA sequences with the NCBI Genebank and RFam database, 187,046 reads of rRNA, tRNA, snRNA, snoRNA and repeat-associated small RNAs were annotated and removed (Figure 2). The remaining 18,882,668 reads, including 9,417,103 and 9,465,565 for the big-size and small-size groups respectively, consisting of 103,234 and 84,208 unique sequences respectively (Additional file 1: Table S1-3), were retained for miRNA analysis.

Due to the lack of whole genome data for *M. amblycephala*, we aligned the selected small RNA sequences to the genome sequence of zebrafish, which is evolutionarily the closest related species with an available sequenced genome, to perform a distribution analysis on a genomic scale using SOAP. For the selection of the computing algorithm, we chose a tolerance of one mismatch for mapping, which resulted in a total of 16,461,503 reads (86.32%) that represent 26,984 unique sRNAs were mapped to the reference genome (Additional file 2: Figure S1).

### Conserved miRNAs in *M. amblycephala*

To identity the known miRNAs in *M. amblycephala*, we compared the data from two libraries to known miRNAs in miRBase 19.0 ( http://www.mirbase.org/). According to the results, we analyzed the base bias on the first position of identified miRNAs with certain length and on each position of all identified miRNAs respectively. Among the 16,411,948 sequences screened in total, 2,711 unique sequences in big-size group were found to be similar to known miRNAs from other species that had previously been deposited in miRBase. Allowing no more than two mismatches between sequences, these miRNAs represented 332 known miRNAs, belonging to 8,171,600 sequences in total in the big-size group. Meanwhile, 2,695 unique sequences dated from small-size group were screened out in the same way, and we identified 343 conserved miRNAs (Additional file 1: Table S1-4). Combining the data from two libraries, a total of 347 unique mature miRNAs were identified, which belong to 123 families, including 326 miRNAs that overlapped between the two libraries, 4 and 15 miRNAs that were detected only in the big-size and small-size libraries, respectively. The reads of these miRNAs were ranged from 1 to 2,649,630, indicating that not only highly expressed miRNAs but also weakly expressed miRNAs were identified by Solexa sequencing. From the 5,406 unique sequences, a total of 60 duplex-like miRNA: miRNA* pairs were obtained, in which the mature miRNAs and miRNA*s (miR-#-5p and miR-#-3p) align to the 5′ and 3′ end regions of the precursors, respectively (Additional file 3: Table S2). Recently, some miRNA* sequences (miR-#-3p) were reported as mature functional miRNAs with abundant expression, and miRNA/miRNA* ratios may vary dramatically in different stages of development [25]. In *M. amblycephala*, it was found that most of the miR-#-3p (miRNA*s) were detected at the same or relatively low expression levels than miR-#-5p. It is suggested that the expression level of miR-#-3p mainly relied on degradation degree and degradation rate, because both strands of miRNA duplex were necessarily produced in equal amounts by transcription. However, some miR-#-3p showed relatively higher expression levels than miR-#-5p (such as miR-206-3p, miR-199-3p, miR-124-3p). The relatively high number of reads of these miRNA*s indicates that it may play a functional role in regulating gene expression. Such a phenomenon has also been described in several previous studies [22,24].

For the two libraries, more than 8 million sequences corresponding to 85.86%, 86.26% of all clean reads were annotated to known miRNAs, respectively, showing that our sRNA libraries were highly enriched with mature miRNAs. The identified sequences being 18 ~ 26 nucleotides in length from two libraries showed a strong bias for U in the first nucleotide (Additional file 4: Figure S2). For the miRNAs that have already been identified and validated, mam-let-7a-5p has the highest expression in both libraries, with 2,393,380 and 2,649,630 sequences in big-size and small-size libraries, respectively. These data were in agreement with other studies of miRNAs [22,26,27], showing that let-7a ranked among the highest expressed miRNAs in the muscle. The mam-miR-1-5p, a muscle specific miRNA, was also abundant in both libraries which has been implicated in the determination of the differentiated state of muscle cells and in myogenesis [28,29]. The mam-miR-122 was also dominant in both libraries belongs to a liver specific miRNA family which is implicated in fatty acid and cholesterol metabolism [30,31]. Previous studies have shown that miR-122 was characterized as the most frequent miRNA isolated in the adult liver, reaching around 70% of all cloned miRNAs [32]. This tissue-specific miRNA is also thought to establish patterns of gene expression and may be responsible for maintaining tissues differentiated states [33,34]. In contrary to let-7a-5p, miR-122, the numbers of some miRNAs (such as miR-132b, miR-183, miR-218b and miR-430) was less than 5 reads in both libraries.

### Novel miRNAs prediction

Since the genome data of *M. amblycephala* is unavailable, the unannotated small RNAs that could be mapped to the zebrafish genome sequences were subjected to novel miRNA prediction analysis of their secondary structure, the Dicer enzyme cleavage site and the minimum free energy using Mireap software ( https://sourceforge.net/projects/mireap/). The approach was used to predict novel miRNAs relies on the phylogenetic conservation of the sequences, and this would cause unidentified novel miRNAs. According to the criteria for miRNAs used in this study, we finally obtained 22 putative novel miRNAs in *M. amblycephala*. Of these novel miRNAs, 14 novel miRNAs were found in both libraries, while 3 novel miRNAs were identified only in the big-size group and 5 novel miRNAs were identified only in the small-size group (Additional file 5: Table S3). Intriguingly, the sequencing frequencies of these novel miRNAs (1,467 in big-size group, 2,130 in small-size group) were much lower than that of conserved miRNAs in the big-size and/or small-size groups’ libraries. The same pattern has also been reported in other species [22,24], which suggests that novel miRNAs are usually weakly expressed while conserved miRNA genes are highly expressed. In addition, the length of the novel miRNA sequences varied from 21 to 23 nt, with a distribution peak at 22 nt (63.89%). The novel miRNAs identified from the two libraries had the same characteristics with the conversed known miRNAs, which have a strong bias for U in the first nucleotide (Additional file 6: Figure S3). Many studies had showed that one notable feature of miRNAs was an overwhelming bias for U at the first position [35,36].

### Differentially expressed miRNAs

**Figure 3:**
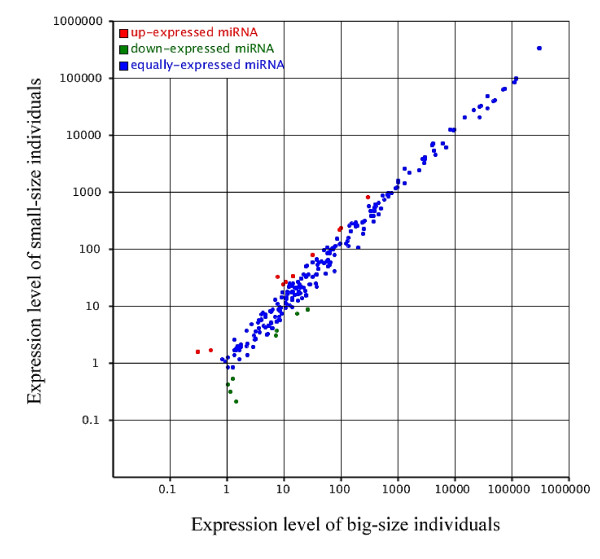
**Scatter plot map for miRNA expression levels in the big-size and small-size groups of *M. amblycephala.*** Each plot represents an individual miRNA. It reflected the proportion of miRNAs that have greater number in the big-size and small-size groups, respectively.

We summarized the common and specific sequences between the big-size and small-size libraries of *M. amblycephala*. Totally, 19,069,714 sRNAs were obtained in total, which included 18,885,300 (189,455 unique) sRNAs in two samples. Thereinto, 100,913 sRNAs that represent 90,385 unique sRNAs were only detected in the big-size group, and 83,501 sRNAs that represent 72,286 unique sRNAs were discovered in the small-size group. The expression of miRNA in two samples was shown by plotting Log2-ratio figure and Scatter Plot (Figure 3). Nineteen conserved miRNAs and 8 novel miRNAs which differentially expressed (*P* < 0.05) between two groups were found (Additional file 7: Table S4). Of the 27 differentially expressed miRNAs, 11 miRNAs and 16 miRNAs had higher expression in the big-size and small-size groups, respectively.

These differentially expressed miRNAs were sequenced at varying frequencies. Some miRNAs such as mam-miR-462, mam-miR-92a, mam-miR-92, were detected with relatively high sequence counts both in the big-size and small-size libraries, although their abundance differed substantially. Subsequently, we compared the differentially expressed miRNAs that have the relatively high reads numbers in both libraries. The expression level of mam-miR-462 (7,665 reads), mam-miR-92a (1,882 reads), mam-miR-92 (2,083 reads) and mam-miR-23b (375 reads) was more than 2 fold higher in the small-size library than that in the big-size library. Remarkably, studies indicated that mam-miR-462 was up-regulated with viral haemorrhagic septicaemia virus (VHSV) infection in zebrafish [36] and rainbow trout [37]. Similarly, mam-miR-92a, mam-miR-92 and mam-miR-23b were associated with proliferation of myeloid cells and biosynthesis of interleukin [38,39]. This result suggests that these miRNAs at high expression level in small-size group may be associated with immunity and disease of fish. In contrast, the sequencing frequencies of some miRNAs, including mam-miR-10b-5p, mam-miR-10d-5p, mam-miR-133b-5p, mam-miR-9b-3p, mam-miR-novel11, mam-miR-novel19, mam-miR-novel21, mam-miR-novel22, mam-miR-novel6, mam-miR-2187-5p, and mam-miR-551, were low in both of the libraries. It is possible that these miRNAs are expressed at low levels in certain cell types and/or under certain conditions.

**Figure 4:**
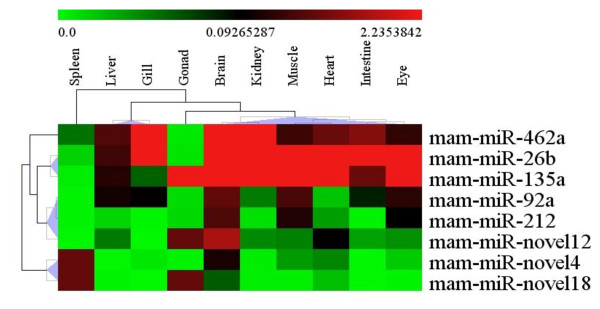
**Heat map showing the 8 differentially expressed miRNAs expression patterns in 10 tissues measured by stem-loop RT-PCR.** Relative expression levels of the 8 differentially expressed miRNAs were measured in terms of threshold cycle value (Ct) and were normalized to 5S rRNA. The expression data were analyzed by hierarchical clustering for both tissues and genes.

Quantifying the differentially expressed miRNAs in the different tissues is an important initial step to investigate the fundamental functions of these miRNAs. We used stem-loop RT-PCR to validate and profile the expression of the differentially expressed miRNAs in 10 tissues of *M. amblycephala*, including brain, heart, intestine, gonad, gill, liver, muscle, kidney, spleen and eye (Figure 4). The results indicated that the expression of conserved miRNAs was significantly higher than that of the novel miRNAs. Intriguingly, mam-miR-novel4 and mam-miR-novel18 were expressed at higher levels than the identified conserved miRNAs in spleen which belong to the key tissue for fish immunity and hematopoiesis [40]. Simultaneously, mam-miR-novel12 and mam-miR-novel18 were expressed at relatively high levels in gonad. These results suggest that mam-miR-novel4, mam-miR-novel12 and mam-miR-novel18 may play roles in the immune regulation and gonad development of *M. amblycephala*. However, these novel miRNAs in the tissues of *M. amblycephala* were expressed at relatively low levels, which coincided with the miRNA profile by sequencing and suggest that novel miRNAs are usually weakly expressed while conserved miRNAs are highly expressed [24,35]. Remarkably, most of the conserved miRNAs are highly expressed in brain, heart, muscle and liver. Mam-miR-462 and mam-miR-26b exhibited high levels of expression in all tissues examined except liver and gonad. Ubiquitous expression of these miRNAs indicates that they may be involved in many fundamental functions. For example, mam-miR-462 is not only associated with type I interferons, which besides their anti-viral effects are known to have major immune regulatory roles in mammals [37], but also involved in vitellogenesis in oviparous animals [36]. Mam-miR-92a and mam-miR-212 showed tissue specific expression patterns, which were highly expressed in brain and heart. Generally, the expression of mam-miR-92a and mam-miR-212 varied substantially among the ten tissues examined.

### Sequence variants and editing of bases in the seed region

**Figure 5:**
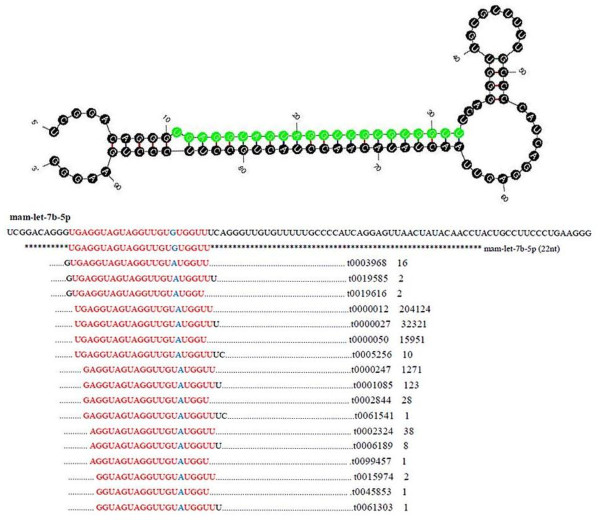
**Details of mam-let-7b-5p isomiRs including sequence count.** A portion of the miRNA precursor, multiple isomiRs with their sequence counts and the pre-miRNA secondary structure with dominant cleavage sites are presented. The most abundant mature miRNAs are indicated by the sequence in green.

Variants of miRNAs, called isomiRs, are commonly reported in deep-sequencing studies. IsomiRs are encoded by the same pre-miRNAs and exhibit sequence variations from the reference miRNAs in miRBase [41]. In this study, such phenomenon was also observed. The sequencing results with the Solexa sequencing found that the majority identified miRNAs showed length and sequence heterogeneity. The length variations in abundant occurred predominantly in the 3′ end of the miRNAs, mainly in the form of missing nucleotides and/or terminal additions of nucleotides. Furthermore, in the majority of the identified miRNAs, frequent nucleotide variations were observed, particularly in the 3′ end, of which some exhibited mismatches to their genomic precursor sequences. The non-templated nucleotides most commonly added at the 3′ terminal ends of mature were most prominently uridine and adenine. A typical example was mam-let-7b-5p, in which the length varied from 18 to 24 nucleotides (Figure 5). The length variations occurred predominantly in the 3′ end of the mam-let-7b-5p, as also observed for other miRNAs identified in this study. The same pattern has previously been reported in pig [26], showing that essentially all miRNAs have length and/or end-sequence variation. These length variations were suggested to arise from variability in Dicer and Drosha cleavage positions or by end-processing, whereas the nucleotide variations are likely to be generated after miRNA maturation. Additional 3′ non-template nucleotides in isomiRs may contribute to miRNA stability and play a key role in miRNA. The studies found isomiRs with 3′ end additions may increase miRNA stability in Drosophila [41] and be involved in the pathogenesis of many human diseases [42], and also attenuated the effectiveness of some specific miRNAs [43,44]. The significance of these nucleotide additions for miRNAs function remains to be determined.

**Figure 6:**
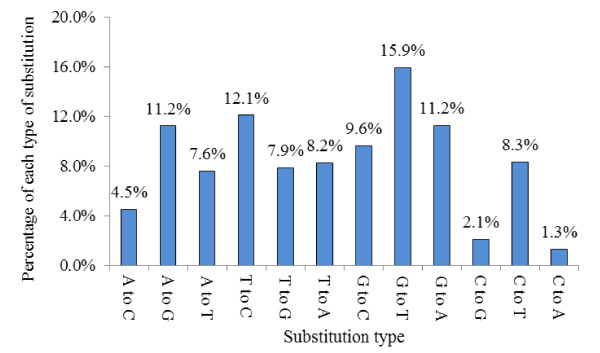
**Histogram displaying the single nucleotide mismatches in the miRNAs seed region sequence when aligning un-annotated sRNAs tags with mature miRNAs from miRBase19.0.** The axis represents the percentage comparing of the observed count of each type to the total count of all of substitution type. Substitutions listed on the abscissa are from DNA (genome) to RNA (small RNA sequence).

Some isomiRs, detected with single nucleotide substitutions including transition and transversion, possibly represent the result of pre-miRNA editing. These end-sequence variations are intriguing as they may allow miRNA variants to perform distinct roles by influencing miRNA/target mRNA hybrid duplex formation. Of the 5,166 single nucleotide substitutions observed in two libraries, the most prominent were G-to-U (15.9%), U-to-C (12.1%), G-to-A (11.2%), and A to G (11.2%), which was similar to several previous studies [26,45]. To perform deep mining of the dataset, we used a computational method to search for base editing in the seed region. In our analysis pipeline, miRNAs which might have the editing in the seed region can be detected by aligning un-annotated sRNA tags with mature miRNAs from miRBase 19.0. The results showed that the total 1,372 mature miRNAs in two libraries displayed single nucleotide substitution in the seed sequence (Figure 6). The edited sites mainly occurred at 5 ~ 8 position (98.2%) in the mature miRNAs. The single nucleotide substitution in the seed sequence was not detected at position 2 ~ 3 in the mature miRNAs. Therefore, the relatively high number of the edited sites occurred at 5 ~ 8 position suggest that the nucleotide at 5 ~ 8 may play a functional role in target mRNA hybrid duplex formation. Many studies indicated that sRNA sequences not perfectly matching the genome are often detected in high-throughput sequencing of the sRNA, and these mismatched sequences are also often attributed to experimental sequencing errors [46]. However, succedent report demonstrated that “sequencing errors” may be the result of post-transcriptional modifications of RNA [47]. Therefore, we presume that the nucleotide substitutions in this study may be attributed to post-transcriptional modifications of RNA. Additionally, it seems that the base editing in seed region of miRNAs in this study invariably correlated with the abundance of miRNAs. Interestingly, there was a positive correlation between the number of the base editing of miRNAs in the seed region and the sequencing abundance, and more future studies are required to functionally validate this conclusion.

### Target prediction of differently expressed miRNAs and KEGG pathway

The identification of miRNA targets is an important step to describe the function of miRNAs well. Computational approaches have been used in many studies as an effective strategy to predict miRNA targets [22-24]. The conventional point of view is that miRNAs regulate target genes by binding to the 3′ UTRs of target mRNAs, and multiple binding sites for multiple miRNAs in 3′ UTRs can strongly enhance the degree of regulation. Recently, many studies have also demonstrated that a large number of miRNA binding sites reside in the 5′ UTR and the coding sequence of mRNAs [48]. A total of 1,901 targets (1,371 unique targets) included 154 EST sequences of *M. amblycephala* were predicted for the 24 highly significant differently expressed miRNAs (*P* < 0.01), which were identified in this study. The predicted targets for the differently expressed miRNAs were shown in Additional file 8: Table S5. Some predicted targets were likely to be targeted by multiple miRNAs at multiple targeting sites. Typically, insulin-like growth factor binding protein 2a (NM_131458) can be targeted by five miRNAs, including mam-miR-462, mam-miR-1-1, mam-miR-122-2, mam-miR-novel 12 and mam-miR-novel 6.

The predicted target genes were classified according to KEGG functional annotations to identify pathways that were actively regulated by miRNA in *M. amblycephala* (Additional file 9: Table S6). Intriguingly, the most over-represented miRNA targets belonged to the metabolic pathways, which is described as a set of complex metabolic networks [49], such as lipid metabolism, carbohydrate metabolism, amino acid metabolism, and energy metabolism. This complex network performs a variety of anabolic and catabolic tasks [50], which serve to transfer part of the chemical energy of the substrate to ATP (or its equivalents) or to NADPH for use in other cell functions, and convert precursor molecules into compounds from which macromolecules, including proteins, complex carbohydrates, nucleic acids, and lipids [51]. Another pathway targeted by the differentially miRNAs was the MAPK signaling pathway, which is a key signaling pathway in skeletal muscle of fish, where its activation is absolutely indispensable for muscle cell proliferation [52]. In fish, the MAPK/ERK can be activated by the insulin-like growth factor-I (IGF-I) in primary cultures of myosatellite cells [53,54], which is a key regulatory hormone that controls growth in vertebrates [55]. Likewise, the insulin signaling pathway was also found to be enriched in our results. Insulin is the most potent anabolic hormone, mediating a wide spectrum of biological responses, including the synthesis and storage of carbohydrates, lipids and proteins and inhibiting their degradation and release back into the circulation [56].

Furthermore, pathways associated with phagosome, biosynthesis of secondary metabolites, tight junction, protein processing in endoplasmic reticulum, purine metabolism, endocytosis, adheren junctions, cell adhesion molecules, regulation of the actin cytoskeleton, focal adhesion, cell cycle and chemokine signaling pathway were all significantly enriched, indicating the role of the differentially expressed miRNAs in the regulation of cell motility, cell proliferation, the cytoskeleton, cell nutrition, communication between cells and the extracellular matrix. Moreover, enriched pathways for neuroactive ligand-receptor interaction, axon guidance and the neurotrophin signaling pathway, suggest that the differentially expressed miRNAs participate in nervous system development and function. On the whole, the results indicated that these differentially expressed miRNAs were involved in regulating growth and metabolism of *M. amblycephala*. It is, however, important for future studies to functionally validate the predictions of these differentially expressed miRNA targets.

## Conclusion

In summary, we identified 347 distinct conserved miRNAs and 22 novel miRNAs from growth related tissues (brain, pituitary, liver and muscle) at different stages of *M. amblycephala* using Solexa sequencing. Our study provides the first large-scale characterization of miRNAs in *M. amblycephala* and miRNA expression in response to the high and low growth rates. The expression levels of these miRNAs displayed a large range, and many of these miRNAs showed differential expression between the big-size and small-size groups. Function annotation of the predicted target genes of the differentially expressed miRNAs revealed a broad range of the metabolic pathways and biosynthesis processes. These findings support the hypothesis that certain miRNAs along with their target genes might be essential in the intricate growth regulation networks, and it will be critical to develop new strategies for the molecular breeding of *M. amblycephala.*

## Methods

### Animals and tissue collection

All experimental animals were derived from offspring of *M. amblycephala* selective population, which were bred in the Ezhou Fish Breeding Base of College of Fisheries, Huazhong Agricultural University. All experimental procedures involving fish were approved by the institution animal care and use committee of the Huazhong Agricultural University. Tissue samples were collected from individuals in big-size group and small-size group, which were selected from the same families, including 3-month-old, 6-month-old, 12-month-old and 18-month-old stages, with 6 individuals from each stage, respectively*.* The fish were anaesthetized in well-aerated water containing the 100 mg/L concentration of tricaine methanesulfonate (MS-222) before tissue collection. Tissue samples including muscle, heart, liver, kidney, gill, spleen, gonad, intestines, pituitary and brain were immediately collected to extract total RNA. The samples were snap-frozen in liquid nitrogen and stored at −80°C.

### Small RNA isolation and cDNA library construction

For Solexa sequencing, tissue samples including brain, pituitary, liver and muscle from the big-size and small-size groups were collected within the same population, respectively. Total RNA was isolated from each sample using Trizol reagent (TaKaRa, Dalian, China) according to the manufacturer’s protocol. RNA quality and quantity was measured using the NanoDrop 2000 (Thermo Scientific, Wilmington, DE, USA). All the samples were standardized to 500 ng/μL, and equal volumes of the tissue samples from different individuals in the same group were combined into one pool.

Two small RNA (sRNA) libraries were constructed for brain, pituitary, liver and muscle tissues from the big-size and small-size groups, respectively. Briefly, small RNAs of 16–30 nt in length were first isolated from the total RNA by size fractionation, and these small RNAs were ligated to with 5′-RNA and 3′-RNA adapters and then reverse transcription PCR using the RT primer was used to create cDNA. Subsequently, a PCR reaction was performed using primers complementary to the two adaptors. The amplified cDNA constructs were purified and sequenced by Illumina/Solexa technology (BGI, Shenzhen, China).

### Sequence data analysis

The resulting images were analyzed using the GENOME ANALYZER PIPELINE Software (version 1.0, Illumina) generating the raw fastq files. The raw reads obtained from Solexa sequencing were processed by summarizing data production, evaluating sequencing quality, calculating the length distribution of small RNA reads. In a fastq format file, one sequence tag (called one “read”) is represented by four lines. The second line is the sequence. The fourth line represents the sequencing quality of this read, which ranges from 0 to 41. This quality will be used in the criteria for filtering out low quality reads. The relationship between sequencing error rate (E) and sequencing quality (*sQ*) is shown in the below formula:

(1) sQ=−10×logE1−Elog10;

(2) $$Y=e^{\frac{\mathrm{sQ}}{-10\times \log10}};$$

(3) $$E=\frac{Y}{1+Y.}$$

Low quality reads were trimmed and reads containing poly A stretches, reads less than 18 nt and adaptor sequences were accurately clipped with the aid of a dynamic programming algorithm. Subsequently, the remaining clean reads were analyzed by BLAST against the Rfam ( ftp://ftp.sanger.ac.uk/pub/databases/Rfam/) database and the GenBank noncoding RNA database ( http://blast.ncbi.nlm.nih.gov/) to annotate rRNA, tRNA, snRNA, repeat associate sRNA, and other ncRNA sequences, and then aligned to exons and introns of mRNA to find the degraded fragments of mRNA in small RNA tags. The common and specific reads of two samples were summarized, including the summary of unique reads and total reads. The clean reads were mapped to the zebrafish genome with a tolerance of one mismatch in the seed sequence by SOAP [57] to analyze their expression and distribution on the genome.

In addition, miRNA identification was performed by comparing the data from two libraries with the known mature miRNAs and the miRNA precursor of all plants and animals in miRBase19.0 ( http://www.mirbase.org/), showing the sequence and count of miRNA families which can be found. Subsequently, we analyzed the base bias on the first position of identified miRNAs with certain length and on each position of all identified miRNAs, respectively. The sequences that are not identical to the conserved miRNAs were used to BLAST against the zebrafish genome to identify potential novel miRNAs using the SOAP software. Sequences with a perfect match or with one mismatch were retained for further analysis. We used Mireap software ( https://sourceforge.net/projects/mireap/) to predict novel miRNA by exploring the secondary structure, the Dicer cleavage site and the minimum free energy of the unannotated small RNA tags which could be mapped to the zebrafish genome. Then the prediction of novel miRNA candidates were summarized, including the base bias on the first position among small RNA candidates with certain length and on each position among all small RNA candidates. Based on this summary, the prediction accuracy could be assessed according to the base bias of known miRNAs.

### Differential expression analysis

To compare the miRNA expression between two libraries to find out the differentially expressed miRNAs, the expression of miRNA in two libraries (big-size group and small-size group) were normalized to obtain the expression of transcripts per million using the following formula: Normalized expression = (Actual miRNA count/Total count of clean reads)*1,000,000. If the normalized expression of a given miRNA is zero, its expression value will be modified to 0.01. If the normalized expression of a given miRNA is less than 1 in both samples, this miRNA is removed in future differential expression analysis. Then, the fold-change and *P*-value were calculated from the normalized expression using the formula. The procedures are shown as below:

$$\text{Fold}\;\text{change}=\text{lo}g_{2}\left(\text{big}-\text{size}\;\text{group}-\mathrm{NE}/\text{small}-\text{size}\;\text{group}-\mathrm{NE}\right)$$

P-value formula:

$$p\left(x\left|y\right)\right)=\left(\frac{N_{2}}{N_{1}}\right){\frac{\left(x+y\right)!}{x!y!{\left(1+\frac{N_{2}}{N_{1}}\right)}^{\left(x+y+1\right)}}}_{D\left(y\geq y_{\max}\left|x\right)\right)=\sum_{y\geq y_{\max}}^{\infty }p\left(y\left|x\right)\right)}^{C\left(y\leq y_{\min}\left|x\right)\right)=\sum_{y=0}^{y\leq y_{\min}}p\left(y\left|x\right)\right)}$$

The N1 and x represent total count of clean reads and normalized expression level of a given miRNA in sRNA library of the big-size individuals’ tissue samples, respectively. The *N2* and *y* represent total count of clean reads and normalized expression level of a given miRNA in sRNA library of small-size’ tissue samples, respectively. When |log2 (big-size group/small-size group) | oupll-size mall0.05, it was be seen as differential expression.

Quantitative stem-loop RT-PCR with SYBR Green PCR Master Mix (Applied Biosystems) was performed to profile the expression levels of the differential expressed miRNAs in 10 tissues. Total RNA from heart, liver, spleen, kidney, muscle, intestine, brain, gill, gonad and eye of *M. amblycephala* was isolated using Trizol reagent (Invitrogen) following the recommendations of the manufacturer, and real-time quantification of miRNAs was performed by stem-loop RT-PCR. Briefly, eight primers of differential expressed miRNAs for stem-loop RT-PCR were designed according to descriptions in prior study [58] (Additional file 10: Table S7). Real-time PCR was carried out on a Rotor-Gene Q real-time PCR Detection System (QIAGEN, Germany) according to the manufacturer’s instructions, and all real-time reactions were performed in triplicate. Relative expression levels of the novel miRNAs were measured in terms of threshold cycle value (Ct) and were normalized to 5S rRNA using the equation 2^-△△Ct^, in which ΔCt = Ct _miRNA_–Ct _5S_.

### MiRNA target prediction

In order to predict the target genes of miRNAs in *M. amblycephala*, a support vector machine (SVM) developed at BGI (Shenzhen, China) was trained to determine the optimal parameters to be use in RNAhybrid. The parameters included helix constraint (2–8), internal loop size (5), bulge loop size (5) and maximum target length (100,000). RNAhybrid predicts potential binding sites for miRNAs in large target RNAs using the principle of finding the most energetically favorable hybridization site between two sequences [59,60]. Considering that the genome references of *M. amblycephala* are not available, we selected the sequences of zebrafish genome and EST sequences of *M. amblycephala* sequenced in our laboratory to predict the target genes with the strategy described in prior studies for target prediction [9,61]. Briefly, the criteria were as follows: 1) no more than four mismatches between sRNA and target (G-U bases count as 0.5 mismatches), 2) no more than two adjacent mismatches in the miRNA/target duplex, 3) no adjacent mismatches in positions 2–12 of the miRNA/target duplex (5′ of miRNA), 4) no mismatches in positions 10–11 of miRNA/target duplex, 5) no more than 2.5 mismatches in positions 1–12 of the of the miRNA/target duplex (5′ of miRNA), and 6) minimum free energy (MFE) of the miRNA/target duplex should be > = 75% of the MFE of the miRNA bound to it’s perfect complement.

### Variants of the miRNAs in *M. amblycephala*

Variants of miRNAs, called isomiRs, are commonly reported in deep-sequencing studies [62]. The Solexa sequencing results in this study revealed that the majority of identified miRNAs showed length and sequence heterogeneity. The nucleotides at position 2–8 of a mature miRNA is known as the seed region (the so-called “miRNA seed”) and this region is highly conserved [63]. The target of miRNA might be different with the change of nucleotides in this region. In our analysis pipeline, miRNAs which might have base edit can be detected by aligning unannotated sRNA tags with mature miRNAs from miRBase19, allowing one mismatch on certain position.

### Availability of supporting data

All small RNA data are available in the NCBI Gene Expression Omnibus database under accession GSE51638. The other supporting data are included as additional files.

## Competing interests

The authors declare that they have no competing interests.

## Authors’ contributions

ZXG and WWM conceived the idea and designed the project. SK, HZ, WL and BC performed the experiments. SK and CZ analyzed the data. SK and ZXG wrote the manuscript. All authors have read and approved the final manuscript.

## Supplementary Material

Additional file 1: Table S1 The basic sequencing information of two small RNA libraries. Click here for file

Additional file 2: Figure S1 Number and distribution of clean reads mapped to the genome sequence of zebrafish. The number of sRNAs on the sense strand of chromosome was shown in blue, whereas the number of sRNAs on the antisense strand of chromosome was shown in red. Click here for file

Additional file 3: Table S2 The conversed miRNAs in *M. amblycephala.* Click here for file

Additional file 4: Figure S2 First nucleotide bias of 18 ~ 26 nt sRNA tags. The numbers above the histogram stand for the tags count in total. Each color in the figure shows the sRNA tags whose first base is a certain base. Click here for file

Additional file 5: Table S3 Predicted chromosomal positions and hairpin structures of the potential novel miRNAs. Click here for file

Additional file 6: Figure S3 Base bias of the novel miRNA candidates of *M. amblycephala* at each position. Click here for file

Additional file 7: Table S4 Abundance and differential expression of miRNAs expressed in both two libraries. Click here for file

Additional file 8: Table S5 The target prediction of differentially expressed miRNAs in *M. amblycephala.* Click here for file

Additional file 9: Table S6 KEGG pathways enriched for targets of the differentially expressed miRNAs in *M. amblycephala.* Click here for file

Additional file 10: Table S7 Primers used in this study for stem-loop real-time PCR. Click here for file
