# Supplementary material for: Identification and characterization of microRNAs involved in growth of blunt snout bream (Megalobrama amblycephala) by Solexa sequencing
Source: BMC Genomics. 2013 Nov 5;14:754. doi: 10.1186/1471-2164-14-754 (PMC3827868; doi:10.1186/1471-2164-14-754)
Supplement: Additional file 2: Figure S1 — Number and distribution of clean reads mapped to the genome sequence of zebrafish. The number of sRNAs on the sense strand of chromosome was shown in blue, whereas the number of sRNAs on the antisense strand of chromosome was shown in red. [file 1471-2164-14-754-S2.docx]

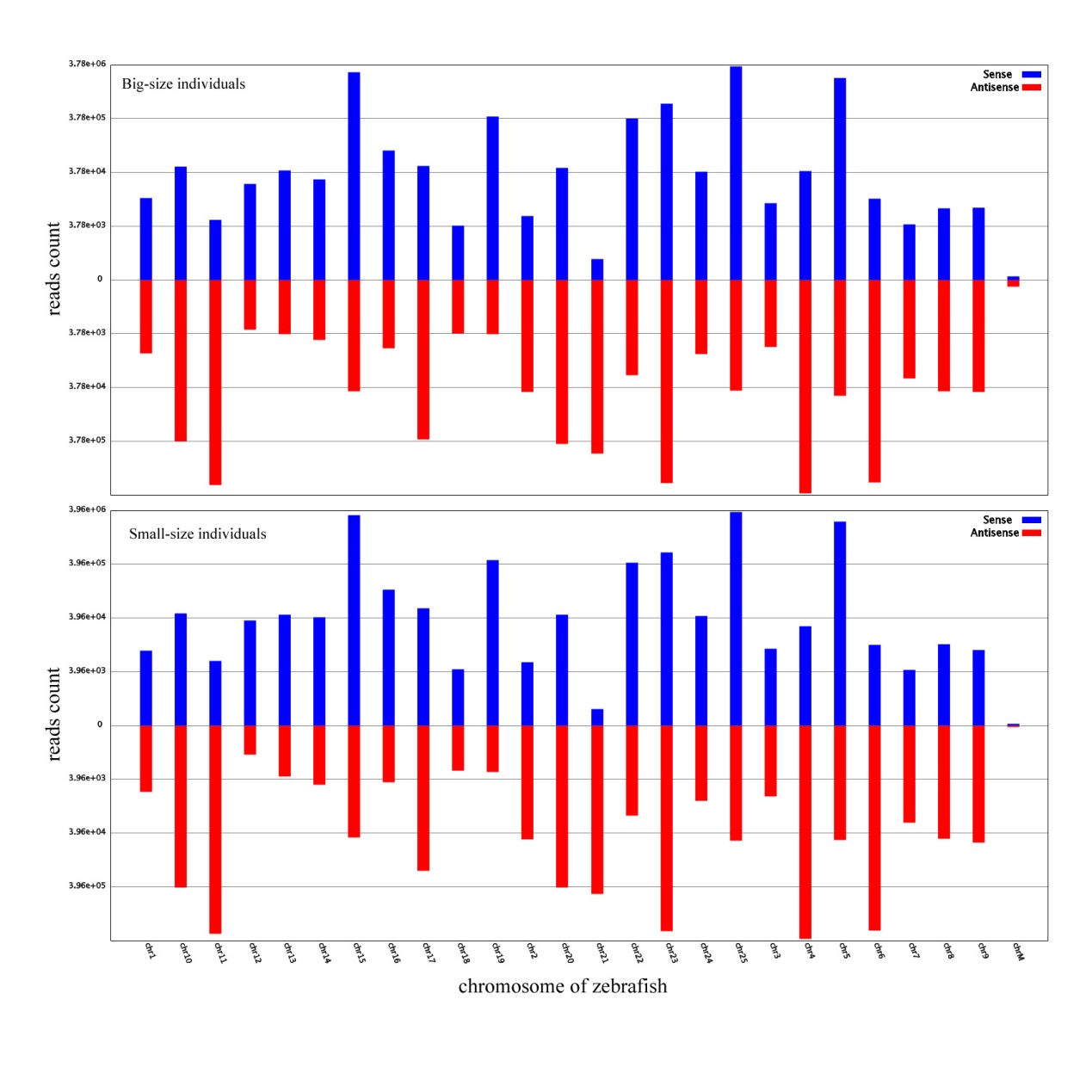


Figure S1 Number and distribution of clean reads mapped to the genome sequence of zebrafish. The number of sRNAs on the sense strand of chromosome was shown in blue, whereas the number of sRNAs on the antisense strand of chromosome was shown in red.
