# Supplementary material for: Identification and characterization of microRNAs involved in growth of blunt snout bream (Megalobrama amblycephala) by Solexa sequencing
Source: BMC Genomics. 2013 Nov 5;14:754. doi: 10.1186/1471-2164-14-754 (PMC3827868; doi:10.1186/1471-2164-14-754)
Supplement: Additional file 4: Figure S2 — First nucleotide bias of 18 ~ 26 nt sRNA tags. The numbers above the histogram stand for the tags count in total. Each color in the figure shows the sRNA tags whose first base is a certain base. [file 1471-2164-14-754-S4.docx]

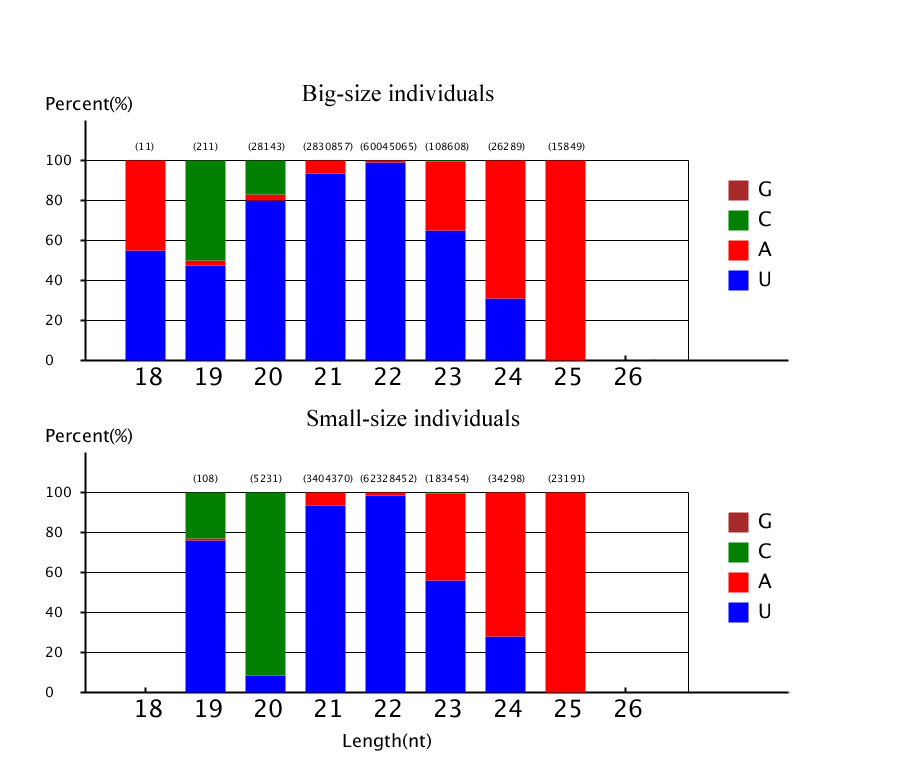


Figure S2 First nucleotide bias of 18~26 nt sRNA tags. The numbers above the histogram stand for the tags count in total. Each color in the figure shows the sRNA tags whose firstbase is a certain base.
