# Supplementary figures and images for: Identification and characterization of microRNAs involved in growth of blunt snout bream (Megalobrama amblycephala) by Solexa sequencing
Source: BMC Genomics. 2013 Nov 5;14:754. doi: 10.1186/1471-2164-14-754 (PMC3827868; doi:10.1186/1471-2164-14-754)

Figure
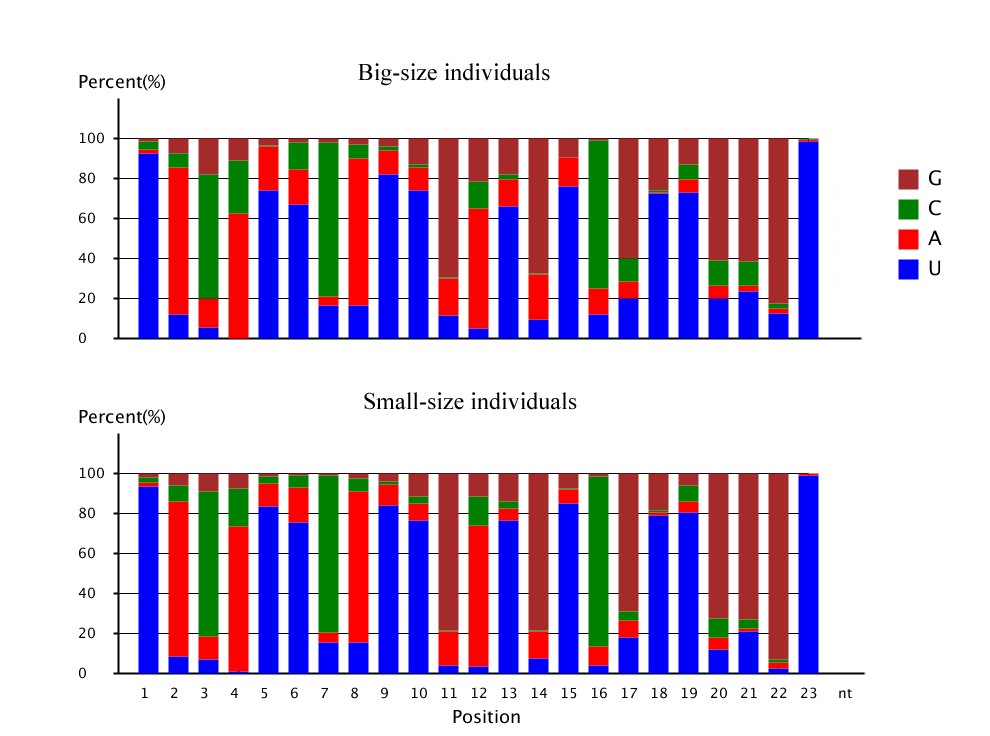
S3 Base bias of the novel miRNA candidates of *M. amblycephala* at each postion

Supplement: Additional file 6: Figure S3 — Base bias of the novel miRNA candidates of M. amblycephala at each position. [file 1471-2164-14-754-S6.docx]
